# Small Bowel Bleeding Due to Vascular Lesions: Pathogenesis and Management

**DOI:** 10.1007/s11894-025-00989-1

**Published:** 2025-06-07

**Authors:** Sunny Sandhu, Jonathan Gross, Jodie A. Barkin

**Affiliations:** Division of Digestive Health and Liver Diseases, Department of Medicine, University of Miami Leonard M. Miller School of Medicine at the University of Miami, 1120 NW 14 th Street, Clinical Research Building, Suite 1188 (D-49), Miami, FL 33136 USA

**Keywords:** Small bowel bleeding, Overt bleeding, Occult bleeding, Gastrointestinal bleeding, Capsule endoscopy, Enteroscopy, Vascular lesions, Angiodysplasia

## Abstract

**Purpose of Review:**

The purpose of this review is to provide a comprehensive review and recent updates in the understanding of the pathogenesis, diagnosis, and management of small bowel vascular lesions.

**Recent Findings:**

Recent terminology has shifted from “obscure GI bleeding” to “small bowel bleeding”, with the former reserved for cases when the source of bleeding is not detected despite a thorough evaluation of the entire GI tract, including the small bowel. Recent diagnostic advances including imaging, video capsule endoscopy (VCE), and deep enteroscopy have allowed for the identification of most small bowel bleeding sources.

**Summary:**

The incidence of small bowel bleeding remains a relatively uncommon event. Vascular lesions remain the most common etiology of small bowel bleeding, with angiodysplasia representing the majority of vascular small bowel lesions. Standard therapeutic approach includes adequate resuscitation and endoscopic evaluation, with consideration of medical therapy (including somatostatin analogues and antiangiogenic agents), endoscopic interventions, radiologic procedures, or surgical therapy in select patients.

## Introduction

Small bowel bleeding (SBB), defined as gastrointestinal (GI) bleeding occurring anywhere from beyond the ligament of Treitz to the ileocecal valve, remains a relatively less common class of GI bleeding, accounting for about 5% of all cases of GI bleeding. Over the recent years, terminology regarding GI bleeding in the small bowel has been revised. “Obscure GI bleeding” (OGIB) had previously been defined as overt or occult GI bleeding of unknown etiology, which persisted despite a negative bidirectional endoscopy with esophagogastroduodenoscopy (EGD) and colonoscopy. Overt OGIB referred to clinically visible melena or hematochezia, whereas occult OGIB referred to cases of presumed GI bleeding in the absence of clinically apparent bleeding (such as iron-deficiency anemia or positive fecal occult blood test) [1, 2]. However, recent advances in diagnostic testing such as radiographic imaging, video capsule endoscopy (VCE), and deep enteroscopy (DE) have allowed for improvements in the ability to identify most SBB sources. Therefore, more recent terminology has shifted from “obscure GI bleeding” to “small bowel bleeding”. Currently, the term OGIB is reserved for cases when the source of bleeding is not detected despite a thorough evaluation of the entire GI tract, including the small bowel. Despite recent advancements in both diagnostic and therapeutic modalities, challenges with accessing and intervening on bleeding small intestinal lesions remain [2, 3].

**Fig. 1:**
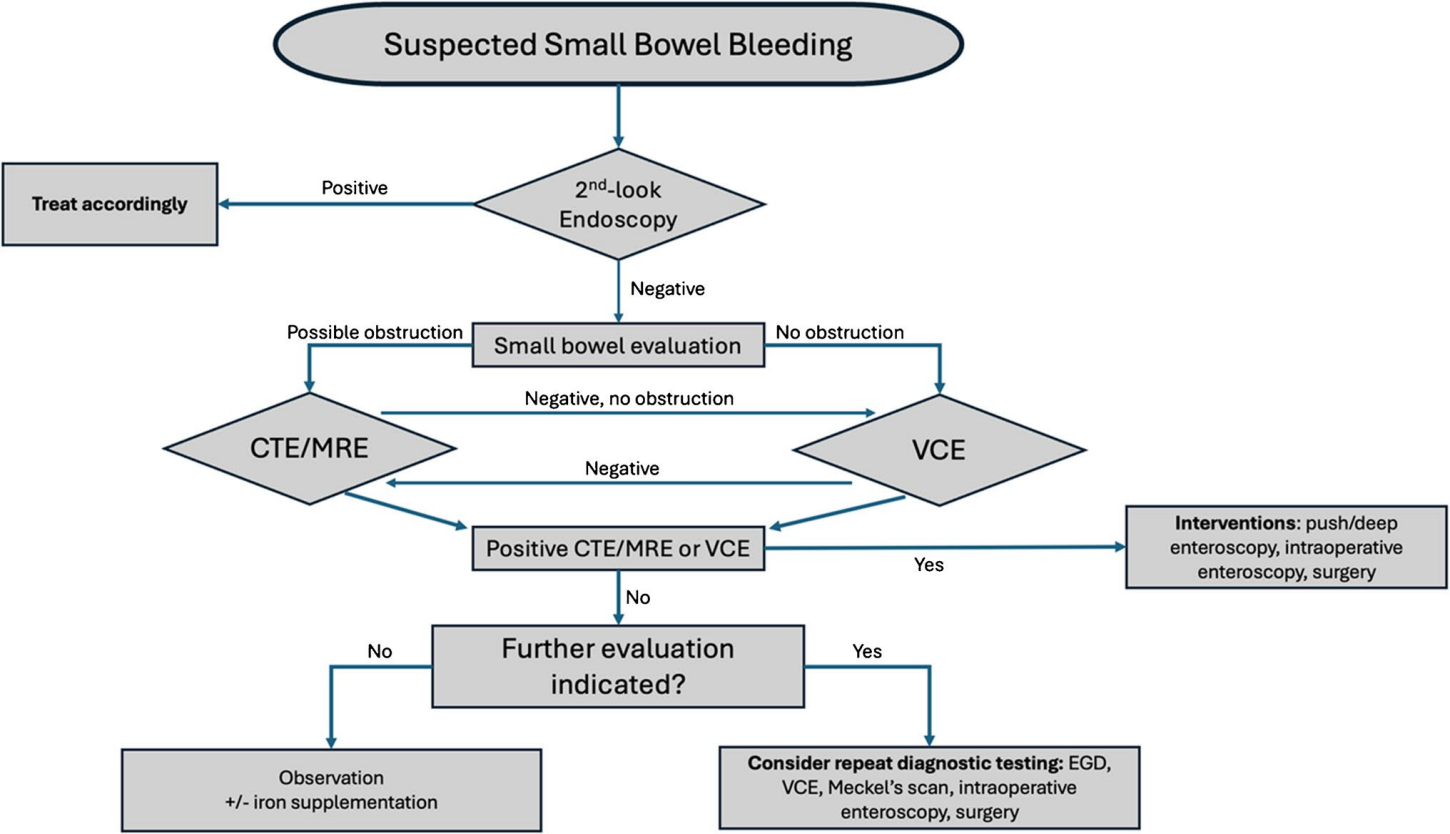
Algorithm for suspected small bowel bleeding. CTE: Computed tomography enterography; MRE: magnetic resonance enterography; VCE: video capsule endoscopy; EGD: esophagogastroduodenoscopy

Although several types of small intestinal lesions have been described as etiologies of bleeding (including inflammatory bowel disease, polyps, and malignancy, amongst others), vascular lesions of the small bowel are the most common [4, 5]. This review will focus solely on SBB due to vascular lesions (Table 1), including pathogenesis and management (Table 2, Fig. 1).

**Table 1:** Vascular Lesions of the Small Bowel

Vascular Lesion	Pathogenesis	Prevalence	Risk Factors	Management
Angiodysplasia	- Chronic/intermittent low-grade obstruction of submucosal veins - Chronic mucosal ischemia - Increased VEGF and FBF expression	- Up to 2.9% in general population - Source of small bowel bleeding in up to 50% of cases	- Age > 50 - Inherited/acquired vWD (AS, LVAD) - Chronic renal failure - NSAID use	- Endoscopic - Surgical - Medications
Dieulafoy lesion	- Mucosal ulcer formation overlying pulsating submucosal artery, subsequent rupture into lumen	- Up to 2% of total cases of GI bleeding	- NSAID use	- Endoscopic - IR embolization - Surgical
Varices	- Dilated portosystemic collateral veins in setting of portal hypertension or prior surgery	- Present in up to 21% of patients with portal hypertension	- Portal hypertension - Abdominal surgery	- Endoscopic: EVL, sclerotherapy - IR: TIPS, BRTO - Surgery
Telangiectasias	-Vascular abnormalities due to systemic or hereditary diseases	-HHT has an estimated prevalence of 1 in 5,000 persons	-Inherited disease with autosomal dominant pattern	-Supportive/ transfusion -Endoscopic

*VEGF* vascular endothelial growth factor; *FBF* fibroblast growth factor; *vWD* Von Willebrand disease; *AS* aortic stenosis; *LVAD* left ventricular assist device; *EVL* endoscopic variceal ligation; *IR* interventional radiology; *TIPS* transjugular intrahepatic portosystemic shunt; *BRTO* balloon-occluded retrograde transvenous obliteration; *HHT* Hereditary hemorrhagic telangiectasia

**Table 2:** Management of Small Bowel Bleeding

Approach	Comments
*MEDICAL*	
Octreotide	Somatostatin analog shown to reduce transfusion need and endoscopies in patients with Ads
Thalidomide	Antiangiogenic agent but with significant potential toxicity and teratogenic side-effects
Bevacizumab	Monoclonal antibody against VEGF-A effective in reducing blood loss in refractory small bowel AD
*ENDOSCOPIC*	
APC	Noncontact method of tissue coagulation by monopolar electrode ionization of argon gas
Electrocoagulation	Monopolar or multipolar devices in which electric current generates heat to achieve hemostasis
Mechanical	Hemostatic clips tamponade by mechanical approximation of tissue
*IR*	For brisk overt bleeding with hemodynamic instability, angiography for selective embolization is recommended. The practice of first performing CTA abdomen & pelvis to identify vessel territory varies by clinical scenario and institution
*SURGICAL*	Small bowel surgical resection is primarily reserved for those needing IOE or in cases of clinically significant suspected SBB in whom VCE, deep enteroscopy, and CTA/angiography have failed to identify or treat the etiology

*VEGF-A* vascular endothelial growth factor A; *APC* argon plasma coagulation; *IOE* intraoperative enteroscopy; *SBB* small bowel bleeding; *VCE* video capsule endoscopy; *CTA* CT angiography

## Etiologies of Small Bowel Bleeding

### Angiodysplasia

Angiodysplasia (AD), also termed as angioectasia or vascular ectasias, refers to abnormally dilated, tortuous, thin-walled blood vessels in the mucosa and submucosa of the gastrointestinal tract. Histologically, the vessels are lined by endothelium alone with minimal or no smooth muscle. Although initially described in 1839, the term AD was first introduced in 1974 by Galdabini when describing abnormal clusters of mucosal vessels in the colon [6]. The prevalence of GI ADs has been reported to be up to 2.9% in healthy patients [7]. The incidence of AD lesions is highest in patients over 50 years old and represents the most common cause of SBB in this age group compared to small bowel tumors, which are the most common SBB etiology in those under 50 years [5, 8]. In a systematic review by Liao et al. including a total of 22,840 procedures, AD was the most common etiology (50.0%) in those with both overt and occult GI bleeding [5].

The pathogenesis for the development of AD is not fully understood; however, several factors have been hypothesized. First, ADs may develop in response to chronic and intermittent low-grade obstruction of submucosal veins at the level of penetration into the muscularis propria, leading to capillary congestion, incompetence of precapillary sphincters and ultimately development of small arteriovenous collaterals [9]. Second, chronic mucosal ischemia has been hypothesized to occur from hypoxia or hypoperfusion in those with underlying cardiac, pulmonary or vascular diseases, which may explain the development in diseases such as aortic stenosis (AS). Third, increased expression of pro-angiogenic factors such as vascular endothelial growth factor (VEGF) and basic fibroblast growth factor have been shown to play a pathogenic role in both the development of AD as well as the risk of bleeding [10, 11]. This association is not only important in the pathogenesis of AD, but also presents a therapeutic role for pharmaceutical therapies discussed later in this review.

Several risk factors for AD have been described to date. Von Willebrand factor (vWF) multimers play a central role in primary hemostasis by acting as a mediator for adhesion and aggregation of platelets to damaged sub-endothelium, contributing to primary hemostasis [12]. Patients with some subtypes of Von Willebrand disease (vWD) have an increased risk of GI bleeding from AD. Low levels of circulating vWF multimers are present in both inherited etiologies such as Type 2a vWD, as well as those with acquired forms including AS. Shear stress of blood flow across the stenotic aortic valve leads to cleavage of vWF multimers, resulting in lower serum levels of vWF multimers [13]. The association with AS was first reported in 1958 and termed ‘Heyde’s Syndrome’ [14]. Several subsequent studies reported an association between AS and recurrent GI bleeding, as well as improvement in patients with AS following aortic valve replacement [13, 15, 16]. Although associations have been made in the literature and hypothesized due to acquired vWF deficiency, a definite causal pathophysiologic relationship has yet to be elucidated but is clinically accepted at this point. Similar pathogenesis of acquired vWF is postulated as the etiology of SBB due to AD in patients with left ventricular assist devices (LVADs) [17]. Additionally, the incidence of AD is higher in those with chronic renal failure, and AD is responsible for most cases of SBB in these patients. The prevalence is significantly higher in those with chronic renal failure at up to 32% compared to 5% in the general population [18, 19]. The pathophysiologic mechanism of AD and chronic renal failure has still not been definitely established; however, several mechanisms have been proposed including uremic platelet dysfunction, chronic intermittent intestinal wall ischemia, aging, and non-steroidal anti-inflammatory drugs (NSAID) and antiplatelet use [20, 21].

### Dieulafoy Lesions

Dieulafoy lesions (DLs) are a relatively rare cause of GI bleeding, and account for up to 2% of total cases of GI bleeding, with up to 95% of DLs found in the stomach. Small bowel DLs are less frequent and mostly described in isolated case reports and case series. DLs are typically a large caliber, tortuous submucosal artery measuring 1–3 mm in diameter, usually in close contact with the mucosal layer [22, 23]. The pathogenesis of DLs is poorly understood and theorized to involve ulcer formation resulting from pulsations at the site of the exposed artery on the surrounding tissue, with subsequent vessel rupture into the lumen. NSAID use has been associated with development of DLs, with ischemia and mucosal injury hypothesized as contributing factors [24, 25]. Clinically, massive and brisk hemorrhage can occur with the erosion of overlying mucosa and the arterial wall. Characteristic findings are an actively spurting pulsatile arteriole arising in a mucosal defect with surrounding normal mucosa without any evidence of inflammation [24]. Although data is limited, most cases have been described in the duodenum, proximal jejunum or distal ileum [22, 26]. Endoscopic identification of DLs can be challenging due to their small size, intermittent exposure and bleeding, and lack of surrounding mucosal injury or inflammatory stigmata [27].

### Varices

Small bowel varices are defined as dilated portosystemic collateral veins, which can occur anywhere throughout the small bowel. They are associated with the various etiologies of portal hypertension or following abdominal surgery where post-operative adhesions contribute to portocaval anastomoses and formation of ectopic varices beneath the intestinal mucosa [28–30]. Ectopic varices are a rare cause of SBB, comprising less than 5% of all cases [31]. Although case series are limited, the presence of small bowel varices may be under-recognized and have been reported in up to up to 21% of patients with portal hypertension [30, 32, 33]. Timely diagnosis and management of small bowel varices can be challenging, as patients can present with brisk, life-threatening bleeding. Management recommendations are based on case series and individual center expertise. Various strategies are available including medical therapies to reduce portal pressures, endoscopic management (variceal ligation or sclerotherapy) that may be challenging depending on location and extent of varices, interventional radiology techniques (transjugular intrahepatic portosystemic shunt [TIPS], balloon-occluded retrograde transvenous obliteration [BRTO], or percutaneous obliteration), and surgery [34].

### Telangiectasias

Hereditary hemorrhagic telangiectasia (HHT), also named Osler-Weber-Rendu Syndrome is a rare disease with various clinical manifestations including arterio-venous malformations which can be present in any organ system [35, 36]. The prevalence of HHT is estimated to be approximately 1 per 5,000 persons [37], but is thought to be underdiagnosed due to absence of obvious clinical symptoms in some individuals. The pattern of inheritance is autosomal dominant, and a variety of mutations have been identified which disrupt transforming growth factor β (TGF-β)-mediated pathways in endothelial cells, leading to abnormal blood vessel development [35]. Mutations in the SMAD4 gene can cause a rare syndrome with overlapping HHT and juvenile polyposis [38], important for the gastroenterologist to recognize due to implications on colonic screening. Gastrointestinal telangiectasias are believed to occur in the majority of patients with HHT [35] and multiple small series looking at gastrointestinal involvement in HHT have reported small bowel telangiectasias in 80–90% of patients with HHT [39–41]. Esophagus, stomach, and large bowel telangiectasias may also develop and have clinical significance, but are less common than small bowel telangiectasias [35]. GI bleeding occurs in approximately 25–30% of patients with HHT, occurring most commonly after age 50 [42]. It is the most common clinical symptom/presentation after epistaxis [43]. Recommendations for management focus on the evaluation of iron deficiency anemia and treatment of acute GI bleeding (though, massive acute GI bleeding is not common from GI telangiectasias from HHT). Endoscopic evaluation should be undertaken in patients with suspected GI bleeding and in whom anemia is disproportionate to level of epistaxis. While argon plasma coagulation (APC) is regarded as the most effective method of endoscopic therapy, if not initially successful, multiple repeat attempts at local endoscopic therapy are not recommended [42]. VCE should be considered when bidirectional endoscopy does not adequately explain the anemia to evaluate for small bowel etiology. Greve et al. suggest that VCE be considered as first-line approach in patients with HHT since it may direct the decision between therapeutic endoscopy or medical treatment in the majority of cases [40]. Endoscopy is advised against in patients with HHT without anemia. Rather, patients over 35 years of age should have annual measurements of hemoglobin or hematocrit [42].

## Diagnosis of Small Bowel Bleeding

### Endoscopic Evaluation

#### Role of 2nd look endoscopy

During initial endoscopic evaluation, it is common to find a lesion within reach of conventional upper and lower endoscopes. However, GI lesions may be missed at initial endoscopy, and in one study, a definite or possible source of GI bleeding was found in up to one fourth to one half of patients only during subsequent evaluation of patients with OGIB [44]. Because of initial endoscopic miss rates, ASGE recommends repeating EGD and colonoscopy before small-bowel evaluation for patients with signs or symptoms consistent with recurrent upper or lower GI sources of bleeding [3]. Factors that weigh in favor of second-look upper endoscopy include recurrent hematemesis, melena, a previously incomplete exam, presence of large hiatal hernias, and a history of NSAID use. Factors that weigh in favor of second-look colonoscopy include recurrent hematochezia or clinical suspicion for missed colon lesions [2, 3]. Second-look colonoscopy is markedly less common than second-look endoscopy in clinical practice. Citing cost-effectiveness, the European Society for Gastrointestinal Endoscopy (ESGE) does not recommend routine second-look endoscopy prior to VCE in patients with suspected small-bowel bleeding or iron deficiency anemia; however, clinically, a second-look endoscopy could be performed at time of endoscopic deployment of video capsule endoscopy [45].

#### Video Capsule Endoscopy

VCE is a noninvasive and safe method for evaluating the entire small bowel and has high positive and negative predictive value in the evaluation of GI bleeding. There is agreement across GI societies that VCE be a first-line diagnostic procedure for small-bowel evaluation and should be performed before deep enteroscopy in most cases [2, 45, 46]. Interventions directed by VCE findings should follow, and if VCE fails to identify a bleeding source, a second VCE may be considered, especially at the time of repeat bleeding [3].

VCE systems include a capsule enteroscope, a sensing system and software for image review and interpretation [47]. The capsule enteroscope may either be swallowed or placed endoscopically primarily into the duodenum or jejunum in cases of postoperative anatomy and progresses through the GI tract by peristalsis until it is excreted. As it progresses, data is wirelessly transmitted from the capsule, which is then processed and displayed to a reader by proprietary software in most currently available VCE platforms with one platform using a “hat and wand” technique to collect the VCE device from the stool, which is then sent for download. Before small bowel VCE, fasting or clear liquid diet for 10–12 h is commonly recommended. There is conflicting data surrounding the role for a full or partial bowel preparation the night before the study [46]. Typically, a clear liquid diet is permitted 2 h after capsule ingestion and a light meal is permitted 4 h after capsule ingestion [46].

Overall diagnostic yield in patients with suspected SBB is cited in the 55% to 62% range [45] and higher in the evaluation of overt SBB [3]. Diagnostic yield is highest when the interval between VCE and the last bleeding episode is as short as possible, and performance within 48–72 h is preferred in inpatients as well as outpatients when possible, with markedly decreased diagnostic yield in those whom VCE is performed over 14 days after a bleeding episode compared to those performed within 14 days of bleeding episode [46]. Patient characteristics that are associated with higher diagnostic yield include presentation with an overt bleed, use of antithrombotic agents, inpatient status, male sex, older age, and liver and renal comorbidities [45].

The main complication of VCE is capsule retention which occurs in approximately 1.5% of patients undergoing evaluation for SBB [2]. A patency capsule (radiopaque nonvideo capsule made of absorbable material) or computed tomography enterography (CTE)/magnetic resonance enterography (MRE) is recommended in patients at high-risk for capsule retention prior to performance of VCE including in those with Crohn’s disease or other history suggestive of/at elevated risk for obstruction or strictures [46], Routine small bowel imaging prior to VCE is not recommended [2]. However, it has been shown that when CTE is performed together with VCE within 30 days, sensitivity is significantly higher than VCE alone, especially for protruding lesions of the small bowel [45].

#### Enteroscopy

Push enteroscopy is an ideal second-look procedure especially when SBB is suspected because of its ability to examine the distal duodenum and proximal jejunum [2]. When SBB is suspected, the diagnostic yield of push enteroscopy ranges from 24 to 56% [3]. As discussed above, a consistent recommendation across society guidelines is for VCE to be the first-line procedure for evaluation of suspected SBB. This potential evaluation should usually follow a negative second-look endoscopy. Because of VCE’s superior diagnostic yield in patients with suspected SBB, push enteroscopy should be utilized as (i) a second-look upper endoscopy [2], (ii) to treat lesions found on VCE and deemed within reach of push enteroscopy [3], or (iii) if proximal lesions are suspected given lower detection rate of lesions in the duodenum and proximal jejunum with VCE, especially in those with history of or known risk-factors for lesions that are common in the proximal small bowel including AD [2].

Deep enteroscopy, also referred to as device-assisted enteroscopy (DAE) includes double-balloon enteroscopy (DBE), single-balloon enteroscopy (SBE), and spiral enteroscopy (SE), and is the technique of choice for therapeutic interventions in the small bowel [48]. DBE (Fujinon Inc, Tokyo, Japan) was introduced in 2001 and comprises an enteroscope, an overtube, and a balloon-pump system [48]. Latex balloons are attached at the tip of the enteroscope and the overtube, and are inflated and deflated by a pump system. As May et al. described, the small bowel is threaded onto the overtube by alternating the inflation and deflation of the balloons, alternating the insertion of the scope and overtube, and pulling back the enteroscope and overtube [49]. Advancement through the small bowel occurs by repeating the above in a series of cycles, referred to as the “push-and-pull technique” [48]. DBE may be performed in either antegrade or retrograde manner, the decision of which should be guided by VCE transit times [46]. Antegrade route should be used for lesions within the proximal two-thirds of the small bowel; retrograde route should be used for lesions within the distal one-third of the small bowel [48]. The diagnostic yield of DBE is reported to be 75% when performed after a positive VCE compared to 27.5% when performed after a VCE with negative result [48]. Further, diagnostic and therapeutic yield has been shown to be higher when DBE was performed earlier [50]. The disadvantages of DBE include length of procedure time, invasiveness, and need for additional personnel for handling the overtube [2]. The overall adverse event rate is cited as approximately 1% in diagnostic and 3–4% in therapeutic DBE [48].

Single-balloon enteroscopy (Olympus, Tokyo, Japan) was introduced in 2007 and has only one balloon at the end of the overtube. Multiple retrospective and prospective trials have compared DBE to SBE and have found similar diagnostic yield, range of available endoscopic therapeutics, and adverse event rates between SBE and DBE [48]. Therefore, ACG recommends any method of deep enteroscopy be used when endoscopic evaluation and therapy is required [2]. The main drawback of SBE is a potentially lower rate of achieving total enteroscopy. Therefore, ASGE recommends DBE as the most effective deep enteroscopy technique for achieving total enteroscopy, when available [48].

Spiral enteroscopy is a two-operator technique introduced in 2008 in which manual rotation of a rigid spiral overtube causes pleating of the small bowel over the enteroscope. The manual spiral enteroscope has since been removed from the market. In 2016, a single-operator motorized power spiral enteroscope (Olympus, Tokyo, Japan) was introduced which accelerated and simplified the procedure [51]. The diagnostic and therapeutic yields for antegrade explorations are similar to those achieved with balloon enteroscopy, with longer insertion length in a shorter procedural time, and higher rates of total enteroscopy compared to traditional SBE and DBE [52]. There remain open questions regarding the need for general anesthesia, learning curve, target population, and the impact of prior major abdominal surgery [45]. Given major complications that subsequently developed with the power spiral enteroscope, it has also been recently removed from the market.

Intraoperative enteroscopy (IOE) is highly sensitive but invasive. Its use should be limited to instances in which enteroscopy cannot be performed such as patients with prior surgeries and intestinal adhesions, or in which traditional device-assisted enteroscopy is unsuccessful at reaching a lesion that is seen on VCE or imaging, or in those with ongoing small bowel bleeding in whom total enteroscopy was not achieved. IOE may be performed orally, rectally, or by enterotomy to evaluate small bowel at the time of laparotomy [2]. Laparoscopic-assisted double balloon enteroscopy has been reported to have faster return of bowel function, shorter time to discharge, and fewer post-operative complications when compared to standard intraoperative enteroscopy [53]. Due to complication and mortality rates when compared to deep enteroscopy, IOE is primarily reserved for patients with recurrent bleeds requiring multiple transfusions or hospitalizations after negative VCE and deep enteroscopy [2].

### Imaging

#### CT Angiography

CT angiography (CTA) aims to identify location, intensity, and cause of a bleed. CTA is highly sensitive (85%−90%), specific (92%), and accurate (94%−95%) for detection and localization of overt GI bleeding [54]. It is noninvasive, rapidly performed, and widely available, making it a preferred test especially in hemodynamically unstable patients with brisk overt GI bleeding [54]. A bleeding rate as low as an estimated 1 mL/min can be detected with modern multidetector CT scanners. Disadvantages include the need for intravenous contrast administration and exposure to ionizing radiation, which is potentially higher than standard CT, because multiple phases of image acquisition are needed for vascular anatomy [54]. The patient must be actively bleeding at the time of the scan in order for contrast extravasation to be detected. Still, blood or clot within the lumen may help localize a bleeding site [2].

#### RBC Scan

Small intestine radionuclide imaging was historically used to diagnose small bowel bleeding, but largely has been replaced by CTA due to false positive tests and poor localization. Technetium-99 m-labeled RBC scintigraphy (RBC scan) is now less commonly performed and considered to be a complimentary imaging method for patients in whom other tests have failed to locate anything or are not available [55]. RBC scan involves intravenous administration of technetium-99 m-labeled red blood cells. The gamma camera then obtains dynamic imaging for a minimum of 1 h. The advantage of RBC scan is high sensitivity due to the ability to detect GI bleeding at a rate of extravasation of 0.1 mL/min in clinical studies and because intermittent bleeding can be captured due to the prolonged nature of the scan. Disadvantages include long imaging time of over 1 h and potentially up to several hours, which requires patients be hemodynamically stable, and imprecise localization of bleeding site as a positive test will give a general quadrant of the abdomen but not localize further [54]. There is wide variation in the reported performance data of RBC scans [2].

#### CT Enterography

CTE is suggested when VCE is negative or contraindicated or when small bowel neoplasm is suspected [3]. The technique requires ingestion of oral contrast to distend the bowel and provide a hypointense background for hyperenhancing pathologic abnormalities that cause GI bleeding [54]. Triphasic CT enterography (tpCTE) uses oral contrast preparation and multiple scanning phases (arterial, enteric, delayed), and was mainly developed to evaluate the small bowel. The implementation of tpCTE is variable and whether it should be performed before or after other diagnostic studies is uncertain [56]. However, approximately 1 in 3 patients with small bowel bleeding have been shown to have a positive finding on tpCTE. In a large cohort study, sensitivity for vascular lesions was 41.9%, representing a high number of actionable targets for DAE or angiography [57].

## Management of Small Bowel Bleeding

### Endoscopic Management

Endoscopic therapies targeting gastrointestinal AD lesions include APC, electrocoagulation, and hemostatic clips. Endoscopic sclerotherapy may be used for varices. Endoscopic injection of epinephrine may be used as adjunctive therapy with one of the subsequently described methods for hemostasis. Lastly, in cases of failure of endoscopic therapies by the methods described below, hemostatic sprays/powders/gels may be considered with variable rates of clinical efficacy.

#### APC

Noncontact method of hemostasis by which inert argon gas is converted to ionized argon gas (plasma) by a monopolar electrode at the tip of the probe which is placed between 2 and 8 mm from bleeding lesion, resulting in tissue coagulation [58].

#### Electrocoagulation

Hemostasis is achieved using electric current passing through a probe to generate heat. With monopolar devices, current passes through the patient and back to the unit via a return pad. With multipolar devices, current is confined to the tissue between the electrodes within the instrument [58].

#### Mechanical Devices

Hemostatic clips effect hemostasis by mechanical approximation of tissue and subsequent tamponade [58].

### Interventional Radiologic Management

For the set of patients who present with hemodynamically unstable suspected SBB, proceeding directly to angiography for selective embolization is recommended before consideration for endoscopic or VCE approaches [3]. Much of the published data on performance of CTA is reported in lower GI bleeding, but the practice is commonly applied to any brisk overt bleeding with hemodynamic instability which may be of small bowel etiology [54]. In some institutions, patients may undergo CTA of the abdomen and pelvis before catheter angiography in order to identify the vessel territory, whereas in other institutions, patients may proceed directly to angiography, depending on the clinical scenario. Once the exact site of bleeding is identified, superselective angiogram of the end vessel supplying the bleeding area is performed followed by microcoil or glue embolization [54]. There is a paucity of outcomes data for angiographic treatment outside of LGIB, for which technical success is cited as greater than 95%, and therefore small bowel angiography success is primarily with extrapolated data [54]. Moreover, the technical and clinical success rates for embolization are significantly worse for the small bowel compared with the large bowel. Navigating the small bowel arterial system with a microcatheter is more complex [59], and embolizing vessels supplying larger territories poses a significant risk of bowel ischemia.

### Medical Management

In severe SBB, transfusion of packed RBCs is essential, especially when medical management and endoscopic management fails. Anticoagulation and/or antiplatelet agents should be discontinued if possible, however, there is no prospective data to show reduced risk of recurrent bleeding from withdrawal of anticoagulation or antiplatelet agents [2].

#### Octreotide

Somatostatin analogs such as octreotide have demonstrated value for treating AD by multiple proposed mechanisms of action [2], including decreased duodenal and splanchnic blood flow, platelet aggregation, and inhibition of angiogenesis [60]. In a 2024 randomized trial by Goltstein et al., patients with ADs and transfusion-dependent anemia who received octreotide required fewer transfusions of blood or iron and fewer per-patient endoscopies than patients who received usual care [60].

#### Thalidomide

Thalidomide is another medical therapy, which has been shown to decrease expression of proangiogenic factors and which has been studied in patients with small bowel AD. Case studies and an earlier pilot open-label controlled trial showed benefit as an antiangiogenic agent [2]. A recent randomized controlled trial by Chen et al. showed that treatment with thalidomide resulted in a significant reduction in bleeding episodes following a treatment period in patients with small bowel AD [61]. Although efficacious, treatment with thalidomide was associated with adverse events and concern was raised by the authors about toxicity especially at higher doses [61]. Thalidomide remains strictly regulated due to its potential teratogenic side-effects in those who are pregnant or may become pregnant.

#### Bevacizumab

Intravenous Bevacizumab, a humanized monoclonal antibody against vascular endothelial growth factor A (VEGF-A), has been shown to be effective in reducing blood loss in refractory small bowel AD. Iyer et al. published data to show efficacy in controlling refractory GI bleeding in patients with hereditary hemorrhagic telangiectasia [62]. Albitar et al. showed similar reduction in transfusion needs, intravenous iron needs, and number of endoscopic procedures in patients with refractory GI bleeding secondary to gastric antral vascular ectasia and small bowel AD [63].

### Surgical Management

The newer and less invasive techniques of VCE and deep enteroscopy have reduced the need for small bowel surgical resection, which is considered an intervention of last resort [2]. VCE performed well in identification of lesions when compared to laparotomy with IOE [64]. In patients with Heyde’s syndrome, aortic valve replacement has been associated with decreased re-bleeding rates [65]. Again, surgery is primarily reserved for those needing IOE or in those with clinically significant suspected SBB in whom VCE, deep enteroscopy, and CTA/angiography have failed to identify or treat the etiology.

## Conclusion

The incidence of SBB remains a relatively uncommon event compared to other sources/locations of GI bleeding. Although previously termed “obscure GI bleeding”, recent diagnostic advances including imaging, VCE, and deep enteroscopy have allowed for the identification of most SBB sources. The term “obscure GI bleeding” should now be reserved for cases in whom a source is not identified despite a thorough evaluation of the entire GI tract, including the small bowel. Vascular lesions, both acquired and systemic/hereditary, remain the most common etiology of SBB, with AD representing the majority of vascular small bowel lesions. Medical therapy includes adequate resuscitation, endoscopic and interventional radiology interventions, and consideration of somatostatin analogues or antiangiogenic agents in patients with recurrent or refractory bleeding. Surgical therapy including bowel resection should be reserved for selective refractory cases, and aortic valve replacement should be considered for those with Heyde’s syndrome.

## Key References

- Gerson LB, Fidler JL, Cave DR, Leighton JA. ACG Clinical Guideline: Diagnosis and Management of Small Bowel Bleeding. *Am J Gastroenterol*. 2015;110(9):1265–1288. 10.1038/ajg.2015.246
- ASGE Standards of Practice Committee, Gurudu SR, Bruining DH, et al. The role of endoscopy in the management of suspected small-bowel bleeding. *Gastrointest Endosc*. 2017;85(1):22–31. 10.1016/j.gie.2016.06.013
- Leighton JA, Brock AS, Semrad CE, et al. Quality Indicators for Capsule Endoscopy and Deep Enteroscopy. *Am J Gastroenterol*. 2022;117(11):1780–1796. 10.14309/ajg.0000000000001903
- Pennazio M, Rondonotti E, Despott EJ, et al. Small-bowel capsule endoscopy and device-assisted enteroscopy for diagnosis and treatment of small-bowel disorders: European Society of Gastrointestinal Endoscopy (ESGE) Guideline—Update 2022. *Endoscopy*. 2023;55(1):58–95. 10.1055/a-1973-3796

## Data Availability

No datasets were generated or analysed during the current study.
